# Behavioral Health and Most-Interested Topics Among Older Adults in Three Aging-in-Community Programs in Florida

**DOI:** 10.1093/geroni/igab046.1345

**Published:** 2021-12-17

**Authors:** Su-I Hou

**Affiliations:** School of Global Health Management & Informatics, University of Central Florida, University of Central Florida, Florida, United States

## Abstract

This study compares behavioral health and most-interested topics among older adults in three aging-in-community (AIC) programs: a university-based lifelong learning program (LLP; 38%), a county neighborhood lunch program (NLP; 29%), and a village program sample (33%) (total n=289). Mean age was 72.4 (SD=8.68) years. Although perceived health was similar (mean=3.76), LLP and village members reported higher quality of life than NLP participants (p=.004). Two-thirds of the participants indicated at least half of their daily plates filled with fruits and vegetables, and at least 10 min. walking in 4.5 days during a typical week. The duration of each walking was lower among NLP members (23 min.), compared with village (31 min.) or LLP members (35 min.) (p=.002). The top three most interested topics were brain health, giving back, and keep community healthy. Older adults in AIC programs were overall healthy and active. Results have implication on tailored program development.

